# UMP kinase activity is involved in proper chloroplast development in rice

**DOI:** 10.1007/s11120-017-0477-5

**Published:** 2018-02-01

**Authors:** Fei Chen, Guojun Dong, Xiaohui Ma, Fang Wang, Yanli Zhang, Erhui Xiong, Jiahuan Wu, Huizhong Wang, Qian Qian, Limin Wu, Yanchun Yu

**Affiliations:** 1 College of Life and Environmental Sciences, Hangzhou Normal University, Zhejiang, China; 2 State Key Laboratory for Rice Biology, China National Rice Research Institute, Zhejiang, China; 3 Institute of Insect Sciences, Zhejiang University, Zhejiang, China

**Keywords:** Chloroplast development, Photosynthetic complexes, Rice (*Oryza sativa* L.), Thylakoid biogenesis, UMP kinase, Yellow leaf mutant

## Abstract

Isolation of leaf-color mutants is important in understanding the mechanisms of chloroplast biogenesis and development. In this study, we identified and characterized a rice (*Oryza sativa*) mutant, *yellow leaf 2* (*yl2*), exhibiting pale yellow leaves with a few longitudinal white stripes at the early seedling stage then gradually turning yellow. Genetic analyses revealed that *YL2* encodes a thylakoid membrane-localized protein with significant sequence similarity to UMP kinase proteins in prokaryotes and eukaryotes. Prokaryotic UMP kinase activity was subsequently confirmed, with YL2 deficiency causing a significant reduction in chlorophyll accumulation and photochemical efficiency. Moreover, YL2 is also light dependent and preferentially expressed in green tissues. Chloroplast development was abnormal in the *yl2* mutant, possibly due to reduced accumulation of thylakoid membranes and a lack of normal stroma lamellae. 2D Blue-Native SDS-PAGE and immunoblot analyses revealed a reduction in several subunits of photosynthetic complexes, in particular, the AtpB subunit of ATP synthase, while mRNA levels of corresponding genes were unchanged or increased compared with the wild type. In addition, we observed a significant decrease (ca. 36.3%) in cpATPase activity in the *yl2* mutant compared with the wild type. Taken together, our results suggest that UMP kinase activity plays an essential role in chloroplast development and regulating cpATPase biogenesis in rice.

**Electronic supplementary material:**

The online version of this article (10.1007/s11120-017-0477-5) contains supplementary material, which is available to authorized users.

## Introduction

Chloroplasts are essential to all plant species, acting as a green factory for carbohydrate production through photosynthesis. They also play an important role in the production of hormones and metabolites, including fatty acids, amino acids, and terpenes (Pogson and Albrecht 2011). In higher plants, chloroplasts develop from undifferentiated proplastids, with the formation of mature photosynthetically active chloroplasts requiring a complex biogenesis process. This process involves import of nuclear-encoded proteins through Toc/Tic complexes, ramping up of pigments, and the establishment of thylakoid membranes (Waters and Langdale 2009; Pfannschmidt et al. 2015). As a semi-autonomous organelle, chloroplasts contain a small autonomous genome that encodes approximately 100 plastid genes, although the majority of several thousand chloroplast proteins are encoded by nuclear genes (Sakamoto et al. 2008). Chloroplast biogenesis and development therefore requires tight regulation of gene expression and assembly of proteins encoded by both plastid and nuclear genes (Mullet 1988). A large number of nucleus-encoded chloroplast proteins have been identified in the model plant Arabidopsis using both forward and reverse genetics (Jarvis and Lopez-Juez 2013; Pogson and Albrecht 2011). These proteins participate in multiple functional processes during chloroplast development, including plastid gene expression, signal transduction, protein synthesis and import, chlorophyll biosynthesis, thylakoid biogenesis, and photosystem assembly (Waters and Langdale 2009). However, despite the discovery of many genes involved in chloroplast development, details of the complex biogenesis of this organelle remain largely unknown.

Recent studies have focused on the molecular mechanism of chloroplast biogenesis by characterizing chlorophyll-deficient mutants in rice (*Oryza sativa* L.), an important cereal crop and model monocot. Rice chlorophyll-deficient mutants exhibit leaf-color phenotypes including albino, chlorina, stripe, virescent, yellow variegated, and zebra (Yoo et al. 2009), all of which are regulated by genes responsible for chlorophyll biosynthesis or chloroplast biogenesis and development (Deng et al. 2014). For example, *chl1* (*chlorina-1*), *chl9* (*chlorina-9*), *ygl1* (*yellow-green leaf1*), *ygl3* (*yellow-green leaf3*), *ygl7* (*yellow-green leaf7*), and *fgl* (*faded green leaf*) mutants specifically disrupt the biosynthesis of chlorophyll in rice (Sakuraba et al. 2013; Tian et al. 2013; Wu et al. 2007; Zhang et al. 2006; Deng et al. 2014; Wang et al. 2010). In addition to these chlorophyll biosynthesis-defective mutants, rice mutants showing altered chloroplast biogenesis and development have also been identified and characterized. For example, *v1* (*virescent-1*), *v2* (*virescent-2*), *v3* (*virescent-3)*, and *st1* (*stripe1*) are temperature-conditional mutants that exhibit abnormal leaf color under low-temperature conditions. *V1* encodes the chloroplast-localized protein NUS1 and functions in chloroplast RNA metabolism during early chloroplast development under low temperature (Kusumi et al. 2011). *V2* encodes a guanylate kinase and is essential for chloroplast differentiation during early leaf development (Sugimoto et al. 2004, 2007), while *V3* and *St1* encode large and small subunits of ribonucleotide reductase (RNR), and are thought to affect DNA synthesis and repair by regulating the rate of deoxyribonucleotide production (Yoo et al. 2009). Moreover, *Young Seedling Albino* (*YSA*) encodes a PPR family protein required for early chloroplast development (Su et al. 2012), while *VYL* (*Virescent Yellow Leaf*) encodes a protein homolog of the Arabidopsis ClpP6 subunit and is also essential for chloroplast development (Dong et al. 2013). *YLC1* (*Young Leaf Chlorosis 1*) encodes a chloroplast-localized protein of unknown function belonging to the DUF3353 superfamily, and is required for chlorophyll and lutein accumulation and chloroplast development during early leaf development (Zhou et al. 2013). Furthermore, *YL1* (*Yellow Leaf 1*), the rice homolog of Arabidopsis EMB1303, encodes a chloroplast-located protein involved in chloroplast development and the regulation of chloroplast ATPase biogenesis (Chen et al. 2016), while *WP1* (*White Panicle 1*) encodes OsValRS2, a Val-tRNA synthetase that plays an essential role in chloroplast development and regulation of ribosome biogenesis (Wang et al. 2016). Collectively, these findings have increased our understanding of the molecular mechanism of chloroplast biogenesis and development in monocotyledonous plants.

Uridine 5′-monophosphate (UMP) kinase catalyzes the ATP-driven conversion of UMP into the corresponding uridine diphosphate (UDP), comprising the first committed step in pyrimidine metabolism (Zhou et al. 1998). UMP kinase is known to play an important role in the regulation of cell proliferation and physiology in both bacteria and yeast (Liljelund and Lacroute 1986; Yamanaka et al. 1992); however, functional studies of UMP kinase in higher plants remain limited (Zhou et al. 1998). Recent genetic analyses of a prokaryotic UMP kinase homologue mutant (*dpt1*) in Arabidopsis suggested a role of UMP kinase in plant chloroplast biogenesis and development (Hein et al. 2009). Moreover, loss of function of *Dpt1* resulted in failure to grow photoautotrophically due to decreased accumulation of PsaA/B transcripts (Hein et al. 2009). Similar effects on chloroplast development were subsequently observed in another prokaryotic UMP kinase homologue rice mutant, *ygl8*, with mutation of *YGL8* resulting in a yellow-green leaf phenotype (Zhu et al. 2016). However, the molecular function of this protein in chloroplast development remains unknown.

In this study, we characterized the rice leaf-color mutant *yellow leaf 2* (*yl2*), which displays pale yellow leaves with a few longitudinal white stripes at the early seedling stage then gradually turns yellow. Map-based cloning revealed that *yl2* is an allelic mutation of the *YGL8* gene (Zhu et al. 2016), a prokaryotic UMP kinase homologue in rice. UMP kinase activity was confirmed and the deficiency of YL2 results in a reduction of chlorophyll accumulation and photosynthesis efficiency. Furthermore, YL2 was targeted to chloroplast thylakoid membranes and found to be essential for the accumulation of AtpA/AtpB subunits of cpATPase, suggesting a possible role in chloroplast development in rice.

## Materials and methods

### Plant materials and growth conditions

The rice (*Oryza sativa*) mutant *yl2* was identified from a mutagenized population of rice ssp. *indica* cv. Shuhui 527 treated with ethyl methanesulfonate (EMS). Shuhui 527 represents the wild type (WT). F2 mapping populations were generated from a cross between the *yl2* mutant and the typical japonica rice variety cv Nipponbare. Rice plants were grown in an experimental field at the China National Rice Research Institute, Hangzhou (latitude 30°26 N, longitude 120°19 E), under natural conditions, or in a growth chamber under a 14 h light (30 °C)/10 h dark (24 °C) cycle.

### Map-based cloning of *YL2*

A total of 733 individual F2 mutant plants screened from a population of *yl2* and Nipponbare were used for genetic mapping. *YL2* was preliminarily mapped to the top of rice chromosome 1 using 20 F2 recessive plants based on 180 microsatellite markers evenly distributed on the 12 rice chromosomes. New genetic markers for fine mapping were developed based on genome polymorphisms between Nipponbare and 93-11 (ssp. *indica*) around the *yl2* locus (Yu et al. 2008). DNA fragments corresponding to candidate genes were amplified by PCR from WT and mutant plants and sequenced to identify the *yl2* mutation. Molecular markers used in this study are described in Supplementary Table 2.

### Plasmid construction and plant transformation

For complementation of the *yl2* mutant, a 7012-bp genomic DNA fragment containing the entire YL2 coding sequence, a 2028-bp promoter region, and an 807-bp downstream sequence was amplified from the genomic DNA of WT plants using primers pCYL2-F and pCYL2-R (Supplementary Table 2) then cloned into the binary vector pCAMBIA1301. The binary construct was then introduced into *Agrobacterium tumefaciens* (strain GV3101) and transformed into *yl2* mutant plants via Agrobacterium-mediated transformation (Hiei et al. 1994).

Construction of a RNAi expression vector was carried out as described previously (Wang et al. 2004). A 340-bp cDNA fragment of YL2 was amplified from cv Nipponbare using the PCR primers YL2-RNAi-F (*Sca*I and *Spe*I) and YL2-RNAi-R (*Bam*HI and *Kpn*I; Supplementary Table 2). The PCR product was fully sequenced and sequentially cloned into the pTCK303 vector to generate a transformation plasmid. This binary construct was then introduced into the Nipponbare background as described above.

### Measurement of chlorophyll contents and photosynthetic characteristics

Chlorophyll contents were examined as described previously (Lichtenthaler 1987). Equal fresh weights (~ 50 mg) of leaf tissues were immersed in 10 mL extract solution (ethanol:acetone:water = 4.5:4.5:1) for 16 h in the dark. The homogenates were centrifuged at 4000×*g* for 5 min and the supernatant was used to determine chlorophyll contents with a Spectrophotometer (Shimadzu UV2450, Japan) at 663 and 645 nm.

Chlorophyll fluorescence measurements were performed with flag leaves of WT and mutant plants at the booting stage. Leaves were dark-adapted for 15 min before measurement using PAM-2500 (Heinz-Walz Instruments, Germany) (Liu et al. 2012). The autofluorescence of leaves from WT and *yl2* mutant seedlings at the 1-week-old was observed using a low-light cooled CCD imaging apparatus (ROPER CA2048B; Roper Scientific). Photosynthetic parameters [photosynthetic rate (Pn), stomatal conductance (Gs), transpiration rate (Tr), and substomatal CO_2_ concentration (Ci)] were determined using an LI-6400 portable photosynthesis system (LI-COR, Lincoln, USA) according to the manufacturer’s instructions.

### Transmission electron microscopy (TEM)

Tissues were collected from first leaves at the top of WT and *yl2* mutant plants at the 1-week-old and booting stages. Samples were prepared as described previously (Chen et al. 2010) and observations performed under a transmission electron microscope (JEOL JEM-1230 EX, Japan).

### Quantitative reverse transcription (qRT)-PCR analysis

Total RNA was isolated using TRIzol reagent (Invitrogen), and first-strand cDNA was synthesized using ReverTra Ace qPCR RT Master Mix with gDNA remover (TOYOBO). qRT-PCR was performed using SYBR Green Real-Time PCR Supermix (Bio-Rad) with a CFX96 Real-Time PCR Detection System (Bio-Rad). The rice *Actin* gene (LOC_Os03g50885) was used as an internal control. Primers used for qRT-PCR are listed in Supplementary Table 2.

### Subcellular localization of GFP proteins

For subcellular localization of YL2, the full-length YL2 coding sequence was amplified using the primers YL2-GFP-F and YL2-GFP-R (Supplementary Table 2) then subcloned into a modified pCAMBIA1300 vector containing a cauliflower mosaic virus (CaMV) 35S::GFP cassette. The resulting fusion construct and empty vector were transferred into rice protoplasts and incubated in the dark overnight before observation according to the protocols described previously (Zhang et al. 2011). GFP fluorescence was observed using a confocal laser-scanning microscope (CLSM) (LSM710, Zeiss, Germany).

### UMP kinase assay

To obtain purified YL2-His protein, full-length YL2 cDNA was amplified using the primers YL2-HIS-F and YL2-HIS-R (Supplementary Table 2) then subcloned into the vector pET44a. The resulting construct and empty vector were expressed in *Escherichia coli* strain BL21. Recombinant YL2-His was purified using Ni-NTA resin (GenScript) and used for kinase assay. The UMP kinase assay was performed as described previously (Yoshida et al. 2012). The 100 μL assay mixture consisted of 50 mM Tris–HCl (pH 7.5), 10 mM MgCl_2_, 1 mM DTT, 0.2 mM UMP, and 10 μM ATP. The reaction was started by the addition of various amounts of purified His-tagged YL2 protein and incubated at 30 °C for 0.5 h. Next, 100 μL of the Kinase-Glo Assay reagent was added to initiate the luciferase reaction. The luminescence intensity was measured after 10 min incubation at room temperature using an ARVO luminometer (PerkinElmer, America).

### Intact chloroplasts and chloroplast subfraction isolation

Intact chloroplasts were isolated from 4-week-old WT seedlings as described previously (Li et al. 2011) with minor modifications. Sixty-gram leaf samples were homogenized in 200 mL ice-cold isolation buffer (50 mM pH 7.8 Hepes–KOH, 0.45 M sorbitol, 2 mM EDTA, 0.1% BSA and 2.5 mM MgCl_2_) then filtered through two layers of Miracloth. The filtrate was transferred to 50 mL centrifuge tubes and softly centrifuged at 1000×*g* for 7 min at 4 °C. Pellets were suspended in 1 mL isolation buffer and layered onto a pre-chilled percoll gradient (10, 40, and 80%, v/v) at 4 °C. Intact chloroplasts fractionated to the 40/80% interface were retrieved and washed in 15 mL HMS buffer (50 mM pH 7.8 Hepes–KOH, 0.33 M Sorbitol, 2.5 mM MgCl_2_) then resuspended in HMS buffer and used for chloroplast subfraction isolation as described previously (Chu and Li 2011).

### 2D-BN/SDS-PAGE and immunoblot analyses

Thylakoid membrane proteins were isolated from 4-week-old WT seedlings as described previously (Zhang et al. 1999). BN-PAGE and two-dimensional analysis were performed as described by Peng et al. (2006), and immunoblot analysis according to our previous study (Chen et al. 2016). Thylakoid proteins were transferred to PVDF membranes (Millipore) after electrophoresis and probed with relevant antibodies specific to PSI subunits PsaA, PsbA (D1), and CP47 (PsbB), cpATPase subunits AtpA and AtpB, and LHCII. Signals were visualized by enhanced chemiluminescence (Invitrogen). Samples were loaded based on an equal chlorophyll content in both BN-PAGE and immunoblot analysis.

### ATPase activity assay

The chloroplast ATPase activity was determined by measuring the amount of inorganic phosphate (Pi) in the reaction by a microcolorimetric method using an ATPase Activity Assay Kit (Sigma-Aldrich). The assay conditions were the same as those described previously (Chen et al. 2016).

## Results

### Phenotype characterization of the *yl2* mutant

**Fig. 1:**
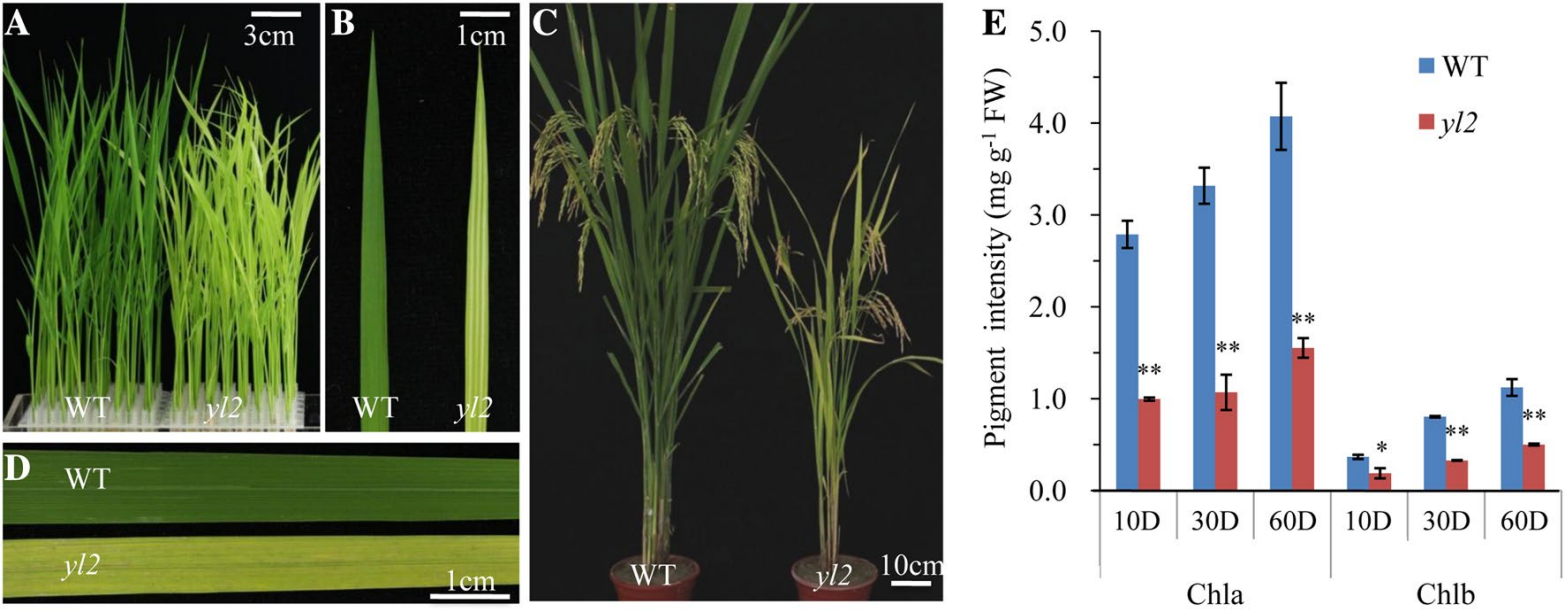
Phenotypic characterization of wild-type (WT) and *yl2* mutant rice (*Oryza sativa*). **a** Phenotypes of 7-day-old WT and *yl2* seedlings cultured in nutrition solution. **b** Enlarged views of leaves from (**a**). **c** Phenotypes of WT and *yl2* plants at the booting stage under field conditions. **d** Enlarged views of leaves from (**c**). **e** Chlorophyll contents of WT and *yl2* mutant leaves at 10, 30, and 60 days after germination (**d**). Data represent means ± SD (*n* = 5). *Significant difference according to the Student’s *t* test at *p < 0.05 and **p < 0.01. *Chla* chlorophyll a, *Chlb* chlorophyll b, *FW* fresh weight

The *yl2* mutant was isolated via EMS mutagenesis of the *indica* rice variety shuhui 527. Mutant plants exhibited pale yellow leaves with a few longitudinal white stripes at the early seedling stage, then turned yellow (Fig. 1a–d). Small or negative effects on major agronomic traits such as plant height, tiller number, seed size, and 1000-grain weight were also observed (Supplementary Fig. 1). Consistent with the phenotype observations, the *yl2* mutant showed reduced chlorophyll contents as well as a reduced chlorophyll a/b ratio throughout development compared to the WT. Chlorophyll a and b contents decreased to approximately 35.7, 32.2, and 38.1% and 51.5, 40.8, and 44.6% of the WT at 10, 30, and 60 days after germination (DAG), respectively (Fig. 1e). Chlorophyll loss in the *yl2* mutant was accompanied by a similar decrease in steady-state chlorophyll fluorescence during illumination (*Fs*), with an equivalent dark fluorescence level (*Fo*) at the seedling stage (Supplementary Fig. 2). These results suggest that the yellow leaf phenotype of *yl2* is the result of a decrease in chlorophyll contents.

### Chloroplast development and photosynthesis are affected in the *yl2* mutant

**Fig. 2:**
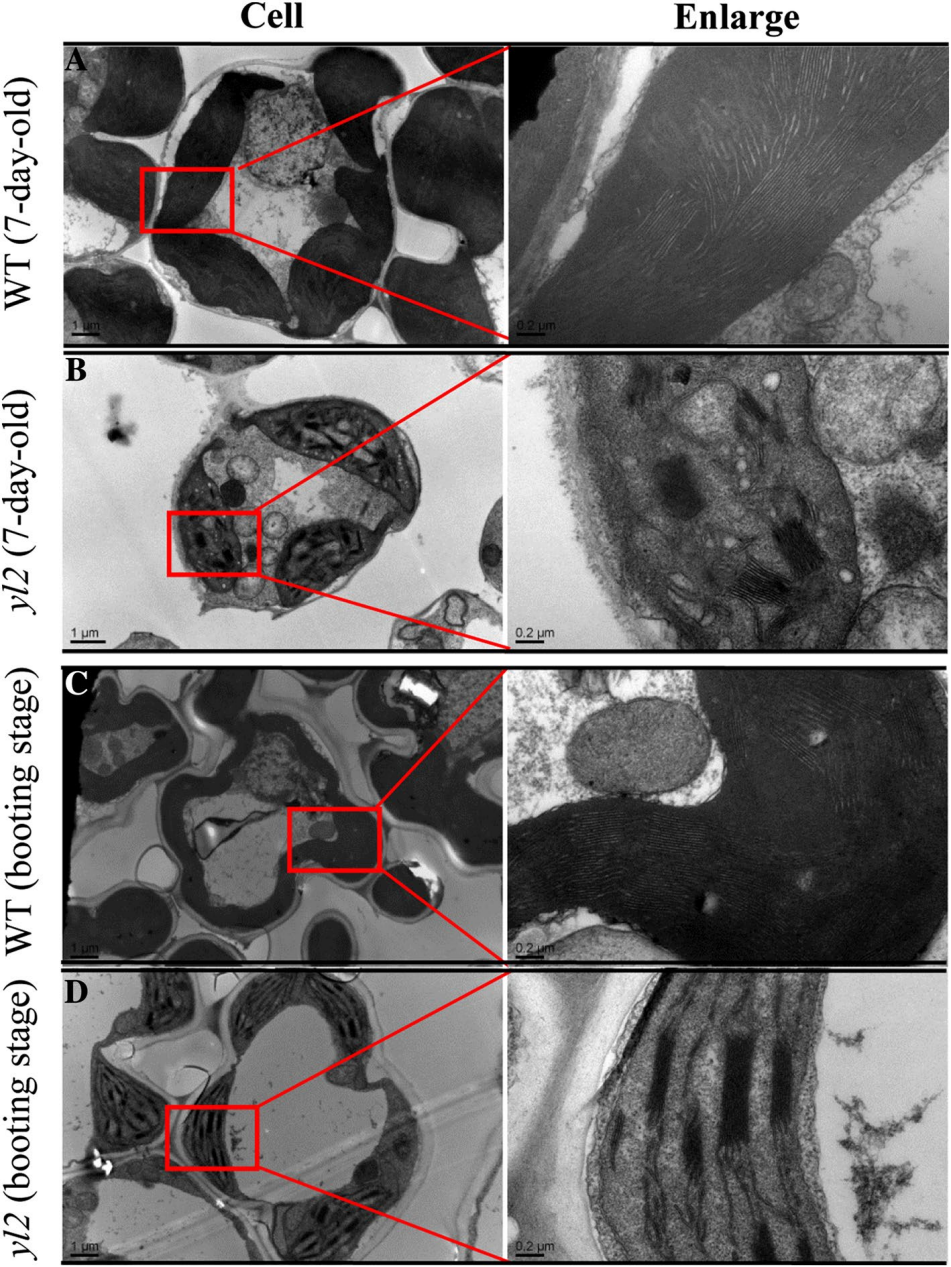
Transmission electron microscope (TEM) observations of wild-type (WT) and *yl2* mutant chloroplasts. Tissues were collected from the first fully expanded leaves of WT and *yl2* mutant seedlings at the 7-day-old (**a**,** b**) and booting (**c**, **d**) stages. Scale bars: 1.0 μm (left) and 0.2 μm (right)

Next, the chloroplast ultrastructure of WT and *yl2* plants at the early seedling (7-day-old) and booting stages was examined using TEM. As shown in Fig. 2a, c, leaf cells from WT plants at the 7-day-old and booting stages contained mature chloroplasts with a normal morphology and well-developed thylakoid structure including stacked grana thylakoids and stroma thylakoids. In contrast, at the early seedling stage, *yl2* mutant chloroplasts exhibited an abnormal morphology, with deformed thylakoid membranes and poorly stacked grana and stroma lamellae compared with the WT (Fig. 2b). Moreover, some chloroplasts from the white striped area contained only rudimentary thylakoids with very few stacked grana (Supplementary Fig. 3). Similar findings were also observed at the booting stage, although disruption was less severe than at the early seedling stage (Fig. 2d). These observations suggest that YL2 plays an important role in chloroplast development, especially thylakoid formation.

To investigate whether the photosynthetic apparatus was also affected in the *yl2* mutant, changes in photosynthetic capacity were examined under field conditions. As shown in Table 1, the *yl2* plants had a lower photosynthetic efficiency, with significant reductions in Pn, Gs, and Tr compared with the WT at booting stage (Table 1). Moreover, loss of YL2 also led to a significant decrease in the maximum efficiency of PSII photochemistry (Fv/Fm) and the effective quantum yield of PSII (ΦII) (reductions of 29.5 and 41.1% compared to the WT, respectively; Table 1). These observations suggest that the photosynthetic capacity is also impaired in the *yl2* mutant.

**Table 1:** Photosynthetic and chlorophyll fluorescence parameters of wild-type (WT) and *yl2* mutant rice (*Oryza sativa*)

	Fv/Fm	Y(II)	Y(NO)	Pn (μmol CO_2_ m^− 2^ s^− 1^)	Gs (mol H_2_O_2_ m^− 2^ s^− 1^)	Ci (μmol CO_2_ mol ^− 1^)	Tr (mmol H_2_O_2_ m^− 2^ s^− 1^)
WT	0.776 ± 0.009	0.645 ± 0.017	0.350 ± 0.014	16.71 ± 1.42	0.838 ± 0.237	299.5 ± 7.7	11.48 ± 0.99
*yl2*	0.547 ± 0.037**	0.380 ± 0.033**	0.539 ± 0.046**	5.20 ± 1.22**	0.529 ± 0.175*	329.4 ± 7.1**	9.19 ± 1.64*

*Fv*/*Fm* maximum quantum yield of PSII, *Y(II)* effective quantum yield of PSII, *Y(NO)* quantum yield of non-regulated energy dissipation in PsII, *Pn* net photosynthetic rate, *Gs* stomatal conductance, *Ci* intercellular CO_2_ concentration, *Tr* transpiration rate

Data represent means ± SD (*n* = 6). Significant difference between the WT and *yl2* mutant according to the Student’s *t* test at *p < 0.05 and **p < 0.01)

### Map-based cloning of *YL2*

**Fig. 3:**
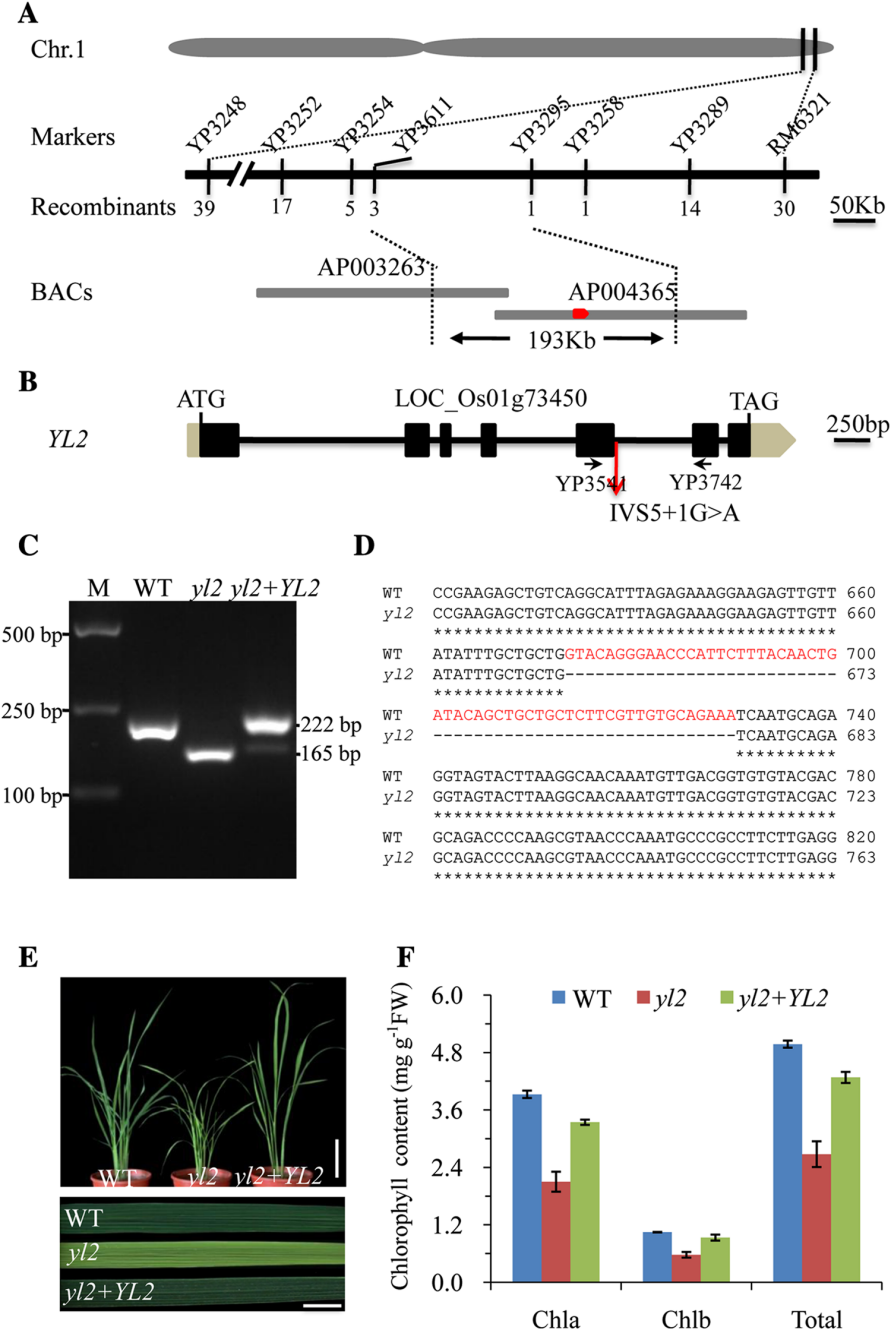
Positional cloning of the *YL2* gene. **a** Map-based cloning of the *YL2* gene. The *YL2* locus was mapped primarily to the long arm of rice chromosome 1 (Chr. 1) between markers YP3248 and RM6321. It was subsequently narrowed to a 193-kb region between YP3611 and YP3295 within the bacterial artificial chromosome (BAC) clones AP003263 and AP004365 using 733 homozygous mutant plants. **b** Structure of the *YL2* gene. Amplification of relevant DNA fragments and sequence comparisons revealed a single base substitution (G to A) in the intron-splicing site of *YL2* (fifth intron). Exons (black boxes), introns (black lines), and the UTR (brown boxes) are indicated. ATG start and TGA stop codons are also shown. **c** Altered splicing of *yl2* transcripts. *YL2* cDNA bands in the wild-type (WT; Line 2), *yl2* mutant (Line 3), and transgenic line showing complemented expression of *YL2* (Line 4), amplified by primers located in two ORFs flanking the mutation site (YP3541 and YP3742). Line 1, molecular weight (M). **d** Alignment of cDNA sequences from the WT and *yl2* mutant. Red markings show a 57-bp miss splicing in the cDNA of *yl2* mutant plants. **e** Phenotypic complementation of the *yl2* mutant after introduction of the YL2 gene. Left: WT, center: *yl2* mutant, right: transgenic line (30-day-old plants, bar: 10 cm). Flag leaves of each line are enlarged in the bottom section of (**e**) to highlight leaf color. **f** Chlorophyll contents of 30-day-old WT, *yl2* mutant and transgenic leaves. Data represent means ± SD (*n* = 5). *Chla* Chlorophyll a, *Chlb* chlorophyll b, *FW* fresh weight

To identify the *YL2* locus, an F2 population was generated from a cross between *yl2* and the japonica variety Nipponbare. The *YL2* locus was initially mapped to the short arm of chromosome 1, between the sequence-tagged site (STS) marker YP3248 and simple sequence repeat (SSR) marker RM6321 (Fig. 3a). For further fine mapping, six new STS markers located between YP3248 and RM6321 were developed. As a result, the *YL2* locus was further delimited to a 193-kb interval between markers YP3611 and YP3295 on BAC (bacterial artificial chromosome) clones AP003263 and AP004365 using 733 F2 recessive plants (Fig. 3a). Within this targeting region, 34 open reading frames (ORFs) were predicted using the Rice Genome Annotation Project (http://rice.plantbiology.msu.edu/). Sequencing analysis revealed a single nucleotide substitution in the intron-splicing site of LOC_Os01g73450 (GT by AT at position + 1 of the fifth intron; Fig. 3b). To verify whether the nucleotide substitution altered splicing of the *yl2* transcripts, cDNA fragments of LOC_Os01g73450 were amplified by primers localized in exons 5 and 6 of both the WT and *yl2* mutant. As shown in Fig. 3c, two PCR products were obtained, one with an expected size of 222 bp in the WT and a shortened product of 165 bp in the *yl2* mutant, confirming altered splicing of the *yl2* transcripts. A new GT site in the fifth extron was recognized by spliceosome, with splicing of 57 additional nucleotides at the end of exon 5, leading to deletion of 19 amino acid and one amino acid transition (I–V) in the putative *yl2* protein (Fig. 3d, Supplementary Fig. 4).

To determine whether the mutated gene was responsible for the *yl2* mutant phenotype, an expression plasmid containing a 7012-bp genomic DNA fragment including the entire coding region of Os01g73450, a 2028-bp upstream sequence, and 807-bp downstream sequence was constructed and transformed into the *yl2* mutant by Agrobacterium-mediated transformation. The presence of normal *YL2* transcripts was confirmed by RT-PCR using primers localized in exons 5 and 6 (Fig. 3c). Phenotypic observations showed that transgenic lines with complementary *YL2* expression in the normal splicing product completely rescued the mutant phenotype (Fig. 3e). In addition, chlorophyll contents in the transgenic positive lines were also restored to WT levels (Fig. 3f). To further confirm that LOC_Os01g73450 is the corresponding *YL2* gene, an RNA interference (RNAi) vector of LOC_Os01g73450 was constructed and introduced into the WT Nipponbare background. Quantitative reverse transcription-PCR (qRT-PCR) analysis showed a significant reduction in LOC_Os01g73450 expression in RNAi transgenic lines compared with the WT (Supplementary Fig. 5c). Moreover, the independent RNAi transgenic lines consistently exhibited an identical phenotype to that of the *yl2* mutant, including yellow leaves, chlorophyll deficiency, and reduced height (Supplementary Fig. 5). Taken together, these findings suggest that the yellow leaf phenotype of the *yl2* mutant is the result of mutation of the LOC_Os01g73450 gene.

### YL2 encodes a functional UMP kinase

**Fig. 4:**
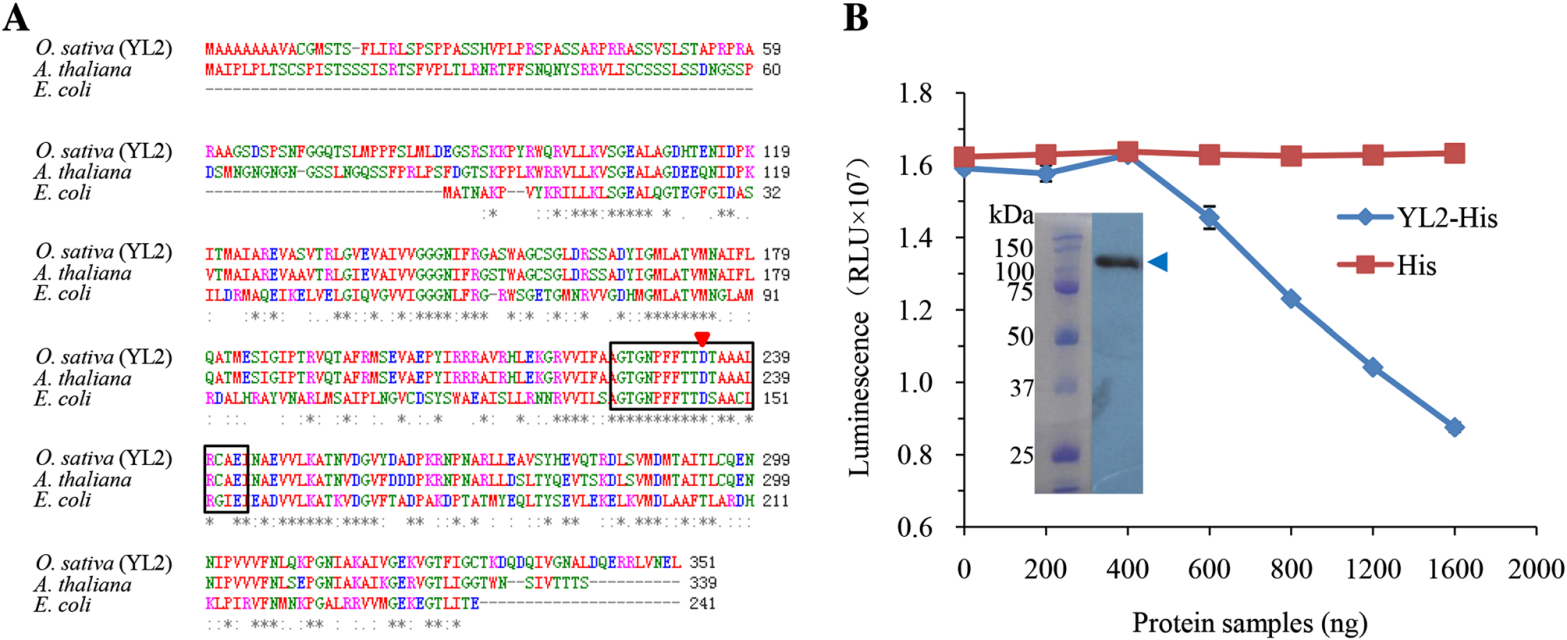
YL2 encodes a UMP kinase. **a** Amino acid sequence alignment of YL2 and representative UMP kinases from *Escherichia coli* (NP_308200) and Arabidopsis (DPT1, At3g18680) using ClustalW (http://www.ebi.ac.uk/Tools/msa/). Color indicates physico-chemical properties of the amino acid, and the symbols below each position in the sequence indicate the amount of conservation (‘*’ exact; ‘:’ conserved substitution; ‘.’ semi-conserved substitution). The boxed regions indicate the mutated amino acid sequences in the *yl2* mutant (Supplementary Fig. 4). The red arrowhead represents the residue (Asp146) required for catalysis (Bucurenci et al. 1998). **b** Kinase assay of YL2 in vitro. UMP kinase activity was assayed at increasing YL2-His fusion protein concentrations using Kinase-Glo reagent (Promega). The inserted image shows the western-blot analysis of the purification of His6-NusA-tagged YL2 (YL2-His, blue arrowhead) from *E. coli*. Cells transfected with empty vector (pET44a, His) served as negative controls

Sequence analysis suggested that *YL2* encodes a 351 amino acid-long protein containing a 58 amino acid putative chloroplast transit peptide at the N-terminus (http://www.cbs.dtu.dk/services/ChloroP/; Supplemental Fig. 4). This protein was identified previously as a nuclear-encoded chloroplast protein which controls leaf color in rice (designated YGL8 Zhu et al. 2016). In this paper we refer to YGL8 as “YL2”. Multiple sequence alignment analysis indicated that YL2 shared significant sequence similarity with other eukaryotic nuclear-encoded UMP kinases and their ancestral prokaryotic UMP kinases and contained functionally important domains found in prokaryotic UMP kinases (Fig. 4a) (Zhu et al. 2016; Hein et al. 2009). Therefore, YL2 may function as a prokaryotic UMP kinase in rice plant. To validate YL2’s biochemical function as a UMP kinase, an YL2 recombination protein tagged with 6 × his at the C-terminus was expressed in *E. coli* and UMP kinase activity examined using Kinase-Glo reagent (Promega). As shown in Fig. 4b, addition of the YL2-His fusion protein decreased levels of RLU, reflecting the amount of residual ATP remaining after the UMP kinase reaction. Meanwhile, the reference reaction (addition of the HIS protein) did not affect RLU levels, confirming that YL2 has prokaryotic UMP kinase activity in vitro.

### YL2 is predominantly expressed in green tissue and is induced during greening

**Fig. 5:**
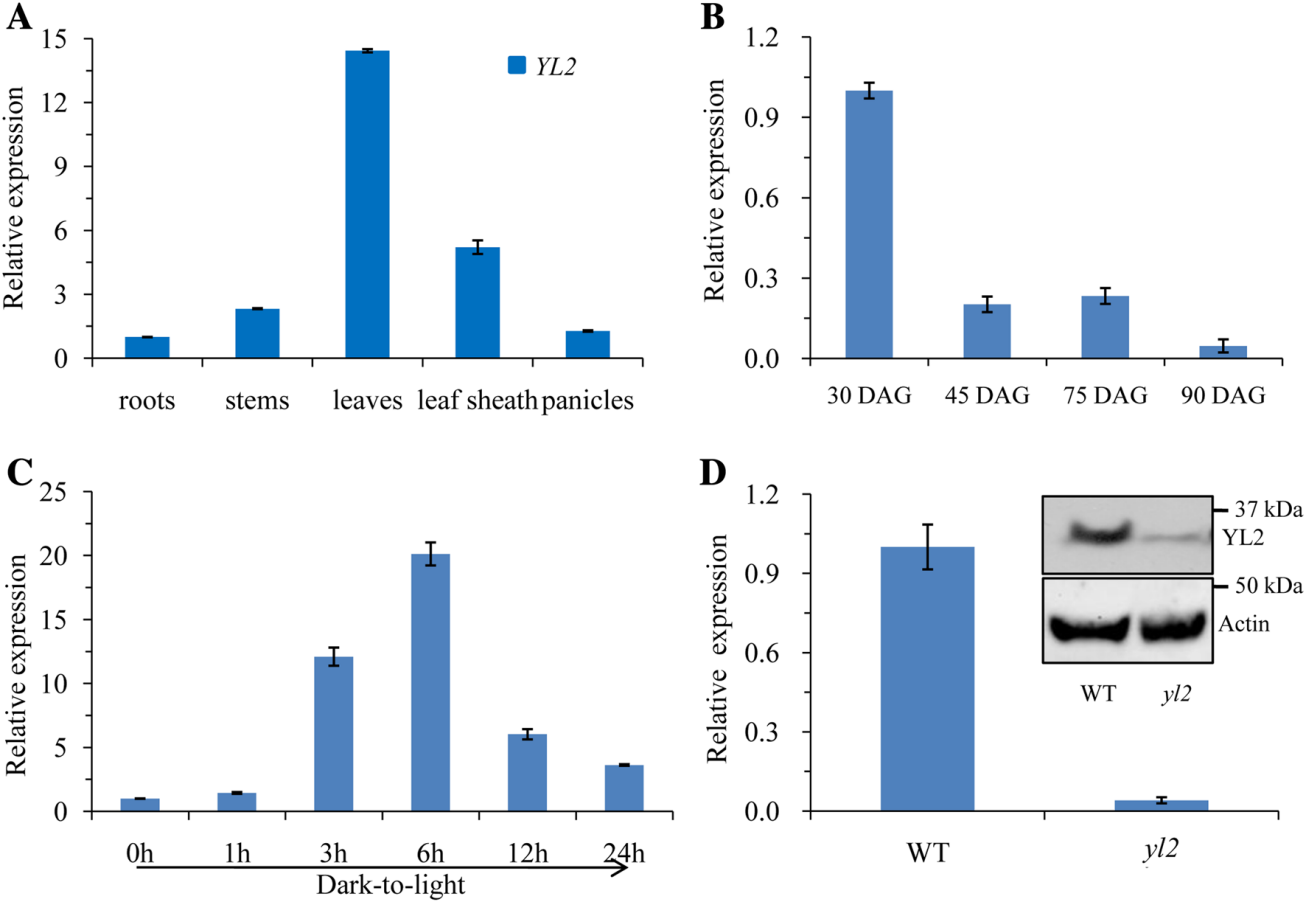
*YL2* expression analysis. **a** qRT-PCR analysis of *YL2* expression in roots, stems, leaves, the leaf sheath, and panicles at the booting stage. **b** mRNA levels of YL2 in leaves at each developmental stage. The YL2 mRNA level in the first leaf of wild-type (WT) plants at 30 days after germination (DAG) was set at 1.0. **c** qRT-PCR analysis of *YL2* expression during greening of etiolated seedlings. WT seeds were germinated and grown in the dark for 10 days. Etiolated seedlings were then illuminated for 0 (control), 1, 3, 6, 12, and 24 h under normal light conditions. **d** Significantly reduced expression levels of YL2 in the *yl2* mutant. The inserted image represents immunodetection of YL2 protein in WT and *yl2* mutant plants. The *YL2* transcript level was normalized to actin gene transcript as a control (LOC_Os03g50885). Data represent means ± SD (*n* = 3)

**Fig. 6:**
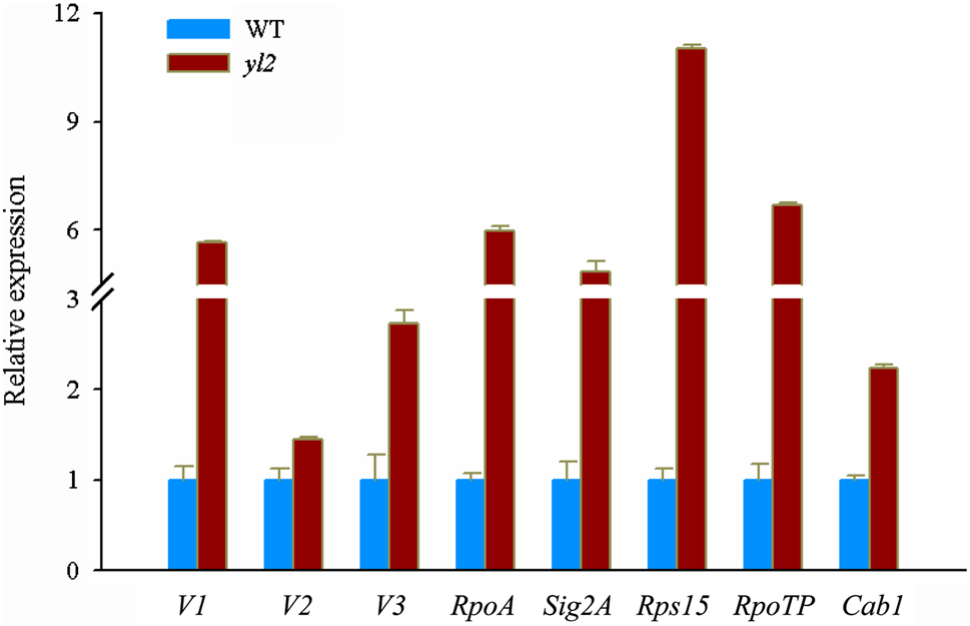
Expression analysis of representative genes involved in chloroplast development and chlorophyll biosynthesis in wild-type (WT) and *yl2* mutant leaves. The relative expression levels of *V1, V2, V3, RpoA, Sig2A, Rps15, RpoTP*, and *Cab1* were analyzed by qRT-PCR and normalized using the Actin gene (LOC_Os03g50885) as an internal control (*mean* ± *SD, n* = 3)

To investigate tissue-specific expression patterns, *YL2* mRNA levels in leaves, leaf sheath, stems, roots, and young panicles of WT plants were examined by qRT-PCR analysis. Expression was highest in the leaves followed by the leaf sheaths, suggesting that *YL2* expression is specific to chloroplast-containing organs (Fig. 5a). Moreover, expression was also influenced by leaf developmental stage. Transcript levels were highest at the seedling stage (30 DAG), but extremely reduced at 45 and 75 DAGs, and weak at 90 DAG (Fig. 5b). Temporal expression patterns of *YL2* during the early stages of leaf greening were therefore examined. Expression increased sharply in the first 6 h after exposure of 7-day-old de-etiolating WT seedlings to light then decreased gradually over time (Fig. 5c). In addition, we found that mRNA and protein levels of YL2 were dramatically decreased (Fig. 5d). We next tested the expression levels of various genes involved in chloroplast development in *yl2* mutant and wild-type seedlings using qRT-PCR analysis. Compared with the WT, transcription levels of genes required for early chloroplast development (Dong et al. 2013), including *Virescent 1* (*nuclear undecaprenyl pyrophosphate synthase 1, NUS1*), *V2* (encoding a guanylate kinase), *V3* (RNR), *RpoTP* (encoding a nucleus-encoded RNA polymerase), *RpoA* (encoding a plastid-encoded RNA polymerase), *Rps15* (*Ribosomal Protein S15*), *Sig2A* (encoding a nucleus-encoded chloroplast sigma factor), and *Cab1* (*Chlorophyll A*/*B binding protein 1*) ,were significantly elevated in the *yl2* mutant (Fig. 6). These results suggest that YL2 plays a role in chloroplast development.

### YL2 protein is localized in the thylakoid membrane

**Fig. 7:**
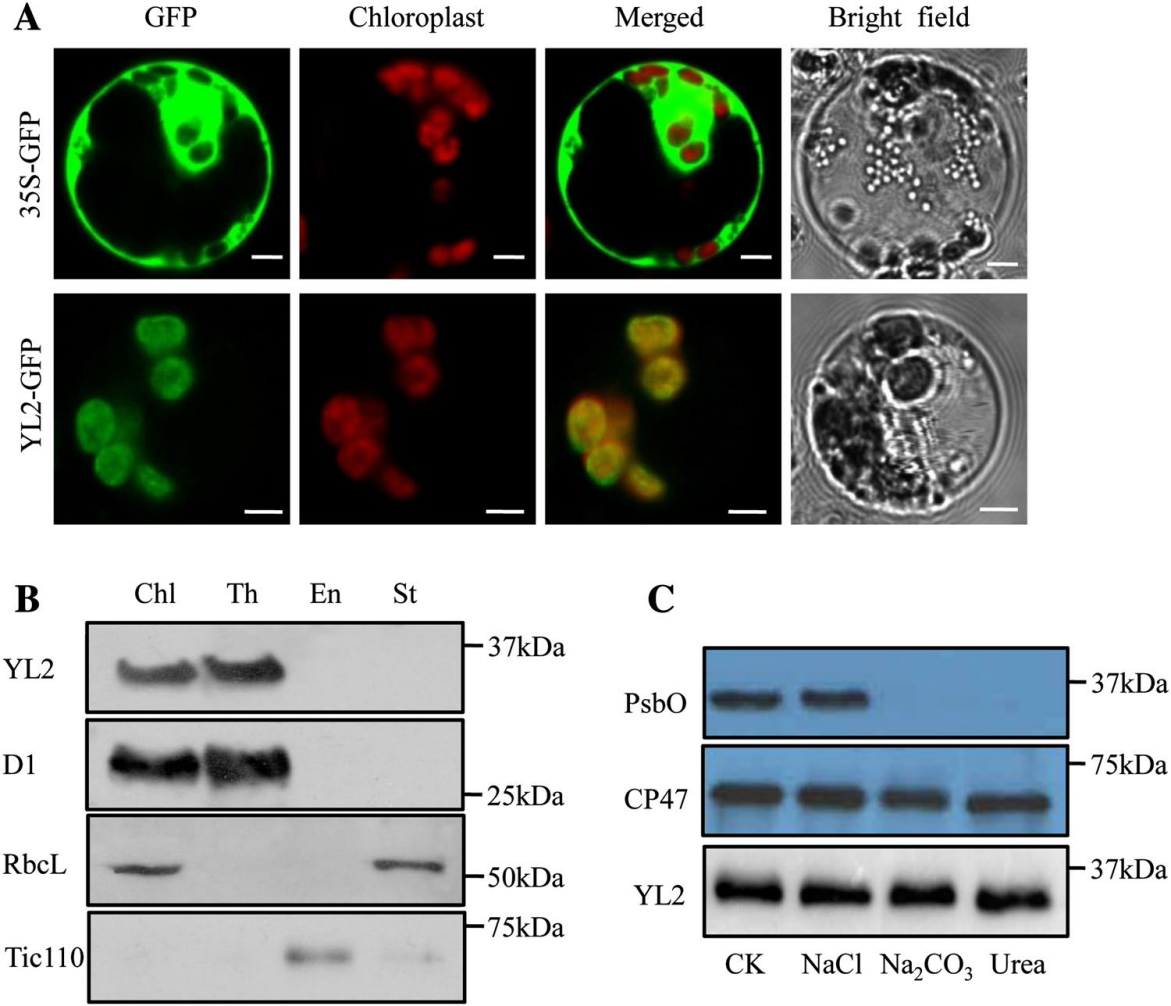
Subcellular localization of the YL2 protein. **a** Subcellular localization of the YL2 protein was determined by GFP assay. 35S::GFP (upper panel) and 35S::YL2-GFP (lower panel) were transiently expressed in rice protoplast cells. Left to right, GFP fluorescence, chlorophyll autofluorescence, merged images, and bright field images. Bar: 5 μm. **b** Suborganelle localization of the YL2 protein in the chloroplast. Intact chloroplasts (Chl) were isolated from WT leaves then separated into thylakoid membrane (Th), envelope (En), and stroma (St) fractions. Proteins were separated by 10% SDS-PAGE, electroblotted onto PVDF membranes then probed with polyclonal antisera against D1 (thylakoid marker), RbcL (stroma marker), Tic110 (envelope marker), and YL2. **c** YL2 represents an intrinsic protein. Thylakoid membrane proteins isolated from WT plants were incubated with 1 M NaCl, 200 mM Na_2_CO_3_, and 6 M urea for 30 min at 4 °C. PsbO (extrinsic membrane protein) and CP47 (intrinsic membrane protein) were used as markers. Membranes without salt treatment were used as a control (CK)

To determine the subcellular localization of YL2, fusion proteins of YL2-GFP (green fluorescent protein) driven by the CaMV 35S promoter (35S::YL2::GFP) were transiently expressed in rice protoplasts. CLSM observations showed free GFP signals dispersed throughout the cell, whereas GFP fluorescence of YL2 was co-localized with the red autofluorescence of chlorophyll, confirming localization in the chloroplasts (Fig. 7a). To further determine the location of YL2 within the chloroplast, the thylakoid membrane, stoma, and envelope fractions of WT chloroplasts were isolated by density gradient ultracentrifugation and analyzed by western blotting. Polyclonal peptide antibodies were raised against the YL2 protein and specific antibody detection of D1, RbcL, and Tic110 in the thylakoid, stroma, and envelope, respectively, was used as a control. As shown in Fig. 7b, the YL2 protein was mainly detected in the thylakoid fraction, with a similar distribution to the thylakoid membrane marker D1 (Fig. 7b). These results suggest that the YL2 protein is localized in the thylakoid membrane.

Sequence analysis revealed that YL2 has two possible transmembrane domains in the regions 128–154 and 166–188, suggesting that YL2 is probably a membrane protein (http://www.cbs.dtu.dk/services/TMHMM/; http://embnet.vital-it.ch/software/TMPRED_form.html, Supplemental Fig. 4). We next tested whether YL2 is an integral or peripheral thylakoid membrane protein. Thylakoid membrane proteins isolated from WT plants were subsequently incubated with different chaotropic reagents (NaCl, CaCl_2_, Na_2_CO_3_, and urea) then subjected to immunoblot analysis. Specific antibody detection of PsbO (extrinsic membrane protein) and CP47 (intrinsic membrane protein) were used as controls. The YL2 protein remained in the membrane fractions even after treatment with the reagents (Fig. 7c), suggesting that YL2 functions as an intrinsic thylakoid membrane-associated protein.

### The *yl2* mutant is defective in the accumulation of AtpA/AtpB subunits of cpATPase

**Fig. 8:**
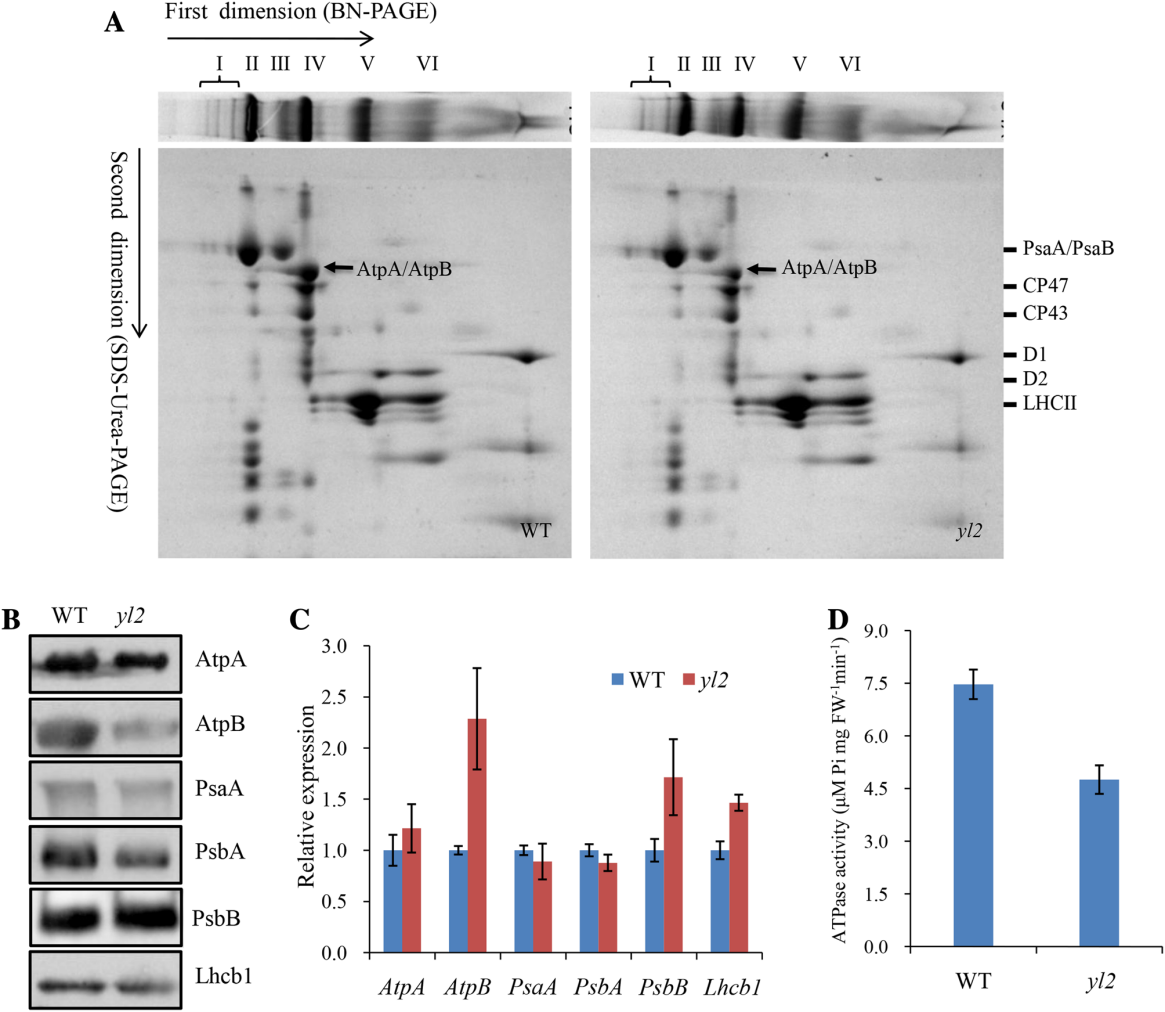
Analyses of thylakoid membrane proteins from wild-type (WT) and yl2 mutant plants. **a** Two-D BN/SDS-PAGE fractionation of thylakoid membrane protein complexes. *I* PSII supercomplex; *II* PSI-PSII dimer; *III* PSI monomer; *IV* PSII core monomer; *V* LHCII trimer; *VI* LHCII monomer. Differentially accumulated protein spots (black arrows) were identified by MALDI-TOF/TOF (Supplementary Table 1). Thylakoid membrane samples extracted from WT and *yl2* mutant leaves. Proteins were loaded on an equal chlorophyll content basis. **b** Western-blot analysis of thylakoid membrane proteins in WT and *yl2* mutant plants. Proteins were separated by 10% SDS-PAGE, electroblotted onto PVDF membranes then probed with specific anti-AtpA, anti-AtpB, anti-PsaA, anti-PsbA (D1), anti-PsbB (CP47), and anti-Lhcb1 antibodies. Proteins were loaded on an equal chlorophyll content basis. **c** Relative expression levels of representative genes encoding thylakoid membrane proteins in leaves from 3-week-old WT and *yl2* mutants. Data represent means ± SD (*n* = 3). **d** ATPase activity in isolated chloroplasts of wild-type and *yl2* mutant plants. Intact chloroplasts were isolated from the leaves of wild-type and *yl2* mutant. Data represent means ± SD (*n* = 4)

The deformed thylakoid structure and localization in the thylakoid membrane suggest a potential role of YL2 in thylakoid membrane formation. Possible changes in the accumulation of photosynthetic complexes in the *yl2* mutant were therefore examined. Thylakoid membranes extracted from the leaves of 4-week-old WT and *yl2* mutant plants were solubilized with dodecyl-*b*-d-maltopyranoside (DM) then separated by BN/SDS-PAGE gels. BN-PAGE gel (first dimension) analysis showed that loss of YL2 results in a slight reduction in the amounts of PSI monomer (band III) and PSII monomer (band IV); however, no significant changes in signal intensities of the remaining three bands were observed compared with the WT (Fig. 8a). Analysis of 2D SDS-urea-PAGE gels after Coomassie blue staining showed a significant reduction in one protein spot separated from band IV (decreased to ~ 40% of the WT, per unit of chlorophyll) (Fig. 8a). The protein represented by this spot was identified by MALDI-TOF/TOF analysis as AtpA/AtpB, core units of the cpATPase complex (Supplementary Table 1). Levels of PSI core subunits PsaA and PsaB and PSII core proteins CP47, CP43, D1, and D2 also decreased slightly in the *yl2* mutant, while accumulation of LCHII was unaltered (Fig. 8a).

Immunoblot analysis was subsequently performed to determine the accumulation of several representative subunits of thylakoid membrane complexes in the WT and *yl2* mutants. Consistent with the results of BN-PAGE analysis, levels of AtpA, AtpB, PsaA, and PsbA were considerably reduced in the *yl2* mutant, with accumulation of AtpB most severely affected. In contrast, levels of PsbB and Lhcb1 were largely unchanged compared to the WT (Fig. 8b). Furthermore, to determine whether the reduced accumulation of thylakoid membrane proteins was the result of reduced transcriptional levels, mRNA levels of corresponding genes were examined in the WT and *yl2* mutant plants using qRT-PCR analysis. As shown in Fig. 8c, expression levels of *AtpA, PsaA*, and *PsbA* were unchanged, while levels of *AtpB, PsbB*, and *Lhcb1* showed a slight increase. These results suggest that YL2 is responsible for thylakoid membrane protein accumulation, especially the AtpB subunit.

### ATPase activity is markedly reduced in chloroplasts of the *yl2* mutant

The above results showed that YL2 is required for the accumulation of core subunits (AtpB/AtpB) of cpATPase. Thus we further determined whether the chloroplast ATPase activity is affected in *yl2* mutant. To this purpose, intact chloroplasts were isolated from the leaves of wild-type and *yl2* mutant plants, and the ATPase activity in isolated chloroplasts was measured on the basis of equal amounts of leaf fresh weight. As shown in Fig. 8d, the chloroplast ATPase activity in *yl2* mutant was markedly reduced to 36.3% of the wide type levels, indicating that the accumulation of functionally active cpATPase complex in *yl2* mutant was significantly affected.

## Discussion

### *YL2* plays an important role in chloroplast development

Although molecular characterization of leaf-color mutants (primarily in Arabidopsis and rice) has allowed the identification of numerous nuclear genes crucial for chloroplast biogenesis, details of this complex process, which relies on thousands of nuclear-encoded proteins, remains largely unknown (Pogson and Albrecht 2011). Isolation of more leaf-color mutants is therefore important to further our understanding of the function of nuclear genes in chloroplast biogenesis and development. In the present study, we characterized a rice yellow leaf mutant, *yl2*, which exhibits pale yellow leaves with a few longitudinal white stripes at the early seedling stage then gradually turns yellow. Mutation of *YL2* resulted in abnormal chloroplast morphology, especially in seedling leaves, leading to diminished chlorophyll fluorescence levels (Fig. 2; Supplementary Fig. 2). Moreover, the plastids did not develop properly in mesophyll cells of yellow areas of *yl2* leaves, and were extremely abnormal in white striped areas, some showing disrupted thylakoid membrane structure (Fig. 2, Supplementary Fig. 3). Chloroplast morphology in the *yl2* mutant seedlings was very similar to that previously described in virescent and stripe rice mutants such as *v3, st1* (Yoo et al. 2009), and *st2* (Xu et al. 2014), which also exhibit disturbed chloroplast biogenesis during early leaf development. Furthermore, we found that mutation of *YL2* significantly enhanced accumulation of transcripts of *V1, V2, V3*, and some chloroplast development-associated genes (*Sig2A, Rps15, RpoTp*, and *RpoA*). These genes encoding the nuclear and plastidic transcription apparatus that are highly coordinated expression at an early stage of chloroplast development (Sakamoto et al. 2008). Similar results were observed in the rice *Virescent Yellow Leaf* (*VYL*) mutant (Dong et al. 2013), and the increased accumulation of these genes could possibly be caused through feedback mechanisms. These results suggest that the loss of function of *YL2* disrupts a necessary process of early chloroplast biogenesis. However, in contrast to virescent and stripe mutants, the white stripes of the *yl2* mutant gradually disappeared with growth and the leaves were never restored to green (Fig. 1c, d). More importantly, the *yl2* mutants showed significantly lower chlorophyll contents, a defective chloroplast morphology, and impaired photosynthetic activity in leaves at the booting stage (Figs. 1e, 2c, d; Table 1). In addition, qRT-PCR analysis revealed strong *YL2* expression at the seedling stage, but relatively low levels at later developmental stages (Fig. 5b). It was therefore speculated that *YL2* is essential for normal chloroplast development, playing an important role in chloroplast biogenesis during early leaf development.

### YL2 protein is localized in the thylakoid membrane and functions as a prokaryotic UMP kinase

Genetic analysis revealed that the *yl2* phenotype resulted from a single nucleotide substitution in the intron-splicing site of LOC_Os01g73450 (IVS5 +1G> A), causing a new splicing site, which led to deletion of 19 amino acids and one amino acid transition (I–V) in the putative YL2 protein (Fig. 3a, b; Supplementary Fig. 4). Sequence analysis revealed that YL2 is a nuclear-encoded prokaryotic UMP kinase-like protein that contains a chloroplast transit peptide (Fig. 4a). The chloroplast location of YL2 was confirmed by subcellular localization of YL2-GFP fusion proteins (Fig. 7a), and subsequent immunoblotting analysis of chloroplast subfractions showed that YL2 is abundant in the thylakoid membrane fraction, functioning as an intrinsic membrane protein (Fig. 7b, c). Moreover, qRT-PCR analysis showed that the *YL2* gene is light dependent and preferentially expressed in green tissues containing chloroplasts (Fig. 5a, c). Taken together, these findings strongly support the suggestion that YL2 is involved in chloroplast biogenesis and development in rice.

Database searches revealed that YL2 shares significant sequence similarity with eukaryotic UMP kinases and their ancestral prokaryotic forms (Zhu et al. 2016). UMP kinase is a crucial enzyme of pyrimidine metabolism (Zhou et al. 1998).The genes encoding UMP kinase have been successfully purified and characterized in both prokaryotes and eukaryotes, including *E. coli, Dictyostelium discoideum, Saccharomyces cerevisiae, Staphylococcus aureus*, and *Sus scrofa* (Serina et al. 1995; Hari Prasad et al. 2012; Liljelund and Lacroute 1986; Okajima et al. 1995; Wiesmuller et al. 1990). However, functional analyses of this enzyme in plants have just begun. To date, only one Arabidopsis cDNA encoding cytosolic UMP kinase activity has so far been cloned, and the specificity of UMP kinase catalytic reactions in Arabidopsis has been characterized (Zhou et al. 1998). Arabidopsis and other eukaryotic UMP kinases share a conserved N-terminal glycine-rich segment, GGPG(S/A)GK, which is important for ATP binding and enzyme activity (Zhou and Thornburg 1998), In contrast, this conserved sequence was absent in YL2 as in prokaryotic UMP kinases (Fig. 4a) (Hein et al. 2009). Sequence alignment further revealed that the YL2 protein contains several conserved residues essential for UMPK/PyrH catalysis in *E.coli* (Fig. 4a) (Bucurenci et al. 1998), confirming its function as a plastid UMP kinase. Moreover, biochemical analysis clarified the UMP kinase activity of the YL2 protein (Fig. 4b). These results suggest that UMP kinase activity in plastids is necessary for chloroplast biogenesis in plants.

A different allelic mutation of the *YL2* gene, *ygl8*, was previously reported (Zhu et al. 2016). Consistent with our observations, mutation of *YGL8* also resulted in a decrease in chlorophyll accumulation and an abnormal grana lamellae structure, causing a yellow-green phenotype throughout development (Zhu et al. 2016). However, the *yl2* mutant has some distinct molecular features compared with the *ygl8* mutant. The expression levels of most nucleus- and plastid-encoded photosynthetic genes were lower in the *ygl8* mutant than that in wild type, but the expression levels of these genes in *yl2* mutant were unchanged or slightly increased compared with WT during early seedling stage (Fig. 8c; Supplementary Fig. 6). The individual phenotype and molecular feature differences between these two allelic mutations at the early seedling stage might be the result of the different mutation sites. In Arabidopsis, mutation of the YL2 homolog At3g18680 (*Dpt1*) resulted in a relatively severe phenotype, with chloroplast developmental defects and seedling death (Hein et al. 2009). There are two possible explanations for this. First, that other plastid UMP kinase-like isoforms exist in rice and exhibit partial functional redundancy with YL2. Second, occurrence of functional specialization of prokaryotic UMP kinase proteins during chloroplast biogenesis processes between monocots and dicots due to the striking differences between Dpt1 and YL2 proteins. Although YL2 shared significant sequence similarity with Arabidopsis Dpt1 protein (Fig. 4a), phylogenetic analysis classified these two proteins into different clades, Dpt1 is in the clade for dicots and YL2 is in the clade for monocots (Zhu et al. 2016). More importantly, Dpt1 is localized in the stroma, whereas our results revealed localization of the YL2 protein in the thylakoid membranes where it acts as an intrinsic membrane protein (Fig. 7). Further analysis of the novel function of YL2 in rice plastids will therefore provide molecular insight into the role of UMP kinase-like proteins in chloroplast biogenesis in higher plants.

### YL2 is responsible for the regulating of cpATPase activity

A previous study in Arabidopsis suggested that the plastid UMP kinase-like protein Dpt1 might have a dual function, contributing to both plastid pyrimidine metabolism and the accumulation of psaA/B transcripts (Hein et al. 2009). UMP kinase participating in the regulation of RNA and protein synthesis also occurs in animals and microorganisms. For example, *pyrH*-encoded UMP kinase plays a direct role in the regulation of the carAB operon in *E. coli* (Kholti et al. 1998). The mutation in *Saccharomyces cerevisiae* gene coding for uridine monophosphokinase causes immediate reduction of pyrimidine triphosphate pools to 10% of the wild-type level as well as significantly lowering total RNA and protein synthesis (Liljelund and Lacroute 1986). In addition, inhibition of PRPP amidotransferase (ATase, which catalyzes the first step in de novo purine synthesis in animal cells) in cultured fibroblasts regulates the rates of DNA and protein synthesis and cell growth (Yamaoka et al. 2001). In this study, we found that the level of the AtpA/AtpB subcomplex of cpATPase was severely reduced in the *yl2* mutant (Fig. 8a). Further immunoblot studies demonstrated that this reduction was mainly due to a significant decrease in AtpB levels (Fig. 8b). Moreover, cpATPase activity is also markedly reduced in *yl2* mutant (Fig. 8d). These results suggest that besides its enzymatic activity, YL2 also plays an important role in the accumulation of cpATPase complex. As we know, cpATPase is an important thylakoid membrane-associated protein complex involved in the light-dependent reactions of photosynthesis (Lyska et al. 2013). It utilizes the proton motive force (pmf) across the thylakoid membrane to drive ATP biosynthesis from ADP and inorganic phosphate (Nelson and Ben-Shem 2004). As we know, ATP is a substrate for UMP kinase enzymic reaction, generally, the mutation of YL2/UMP kinase may disturb the balance of ATP/ADP pool in chloroplasts, and subsequently impair the normal biogenesis of cpATPase.

In conclusion, this study documented the identification and characterization of a thylakoid protein, YL2, which encodes a UMP kinase-like protein in rice. YL2 was found to have prokaryotic UMP kinase activity, playing an important role in the accumulation of AtpA/AtpB subunits of cpATPase complex, which is essential for proper chloroplast development in rice.

## Electronic supplementary material

Below is the link to the electronic supplementary material.

Supplementary material 1 (PDF 528 KB)
