# Supplementary material for: UMP kinase activity is involved in proper chloroplast development in rice
Source: Photosynth Res. 2018 Feb 1;137(1):53–67. doi: 10.1007/s11120-017-0477-5 (PMC5999181; doi:10.1007/s11120-017-0477-5)
Supplement: Supplementary file 1 — Supplementary material 1 (PDF 528 KB) [file 11120_2017_477_MOESM1_ESM.pdf]

UMP Kinase Activity Is Involved in Proper Chloroplast Development in Rice

Fei Chen[1, †], Guojun Dong[2, †], Xiaohui Ma[1], Fang Wang[3], Yanli Zhang[1], Erhui Xiong[1], Jiahuan Wu[1], Huizhong Wang[1], Qian Qian[2], Limin Wu[1*], and Yanchun Yu[1,*]

[1]College of Life and Environmental Sciences, Hangzhou Normal University, Hangzhou 310036, China

[2]State Key Laboratory for Rice Biology, China National Rice Research Institute, Hangzhou 310006, Zhejiang, China

[3]Institute of Insect Sciences, Zhejiang University, Hangzhou 310058, China

[*]Correspondence:

Limin Wu,

Email: lmwu2006@aliyun.com

Yanchun Yu,

Email: ycyu@hznu.edu.cn;

Tel: +(86) 571 28866569

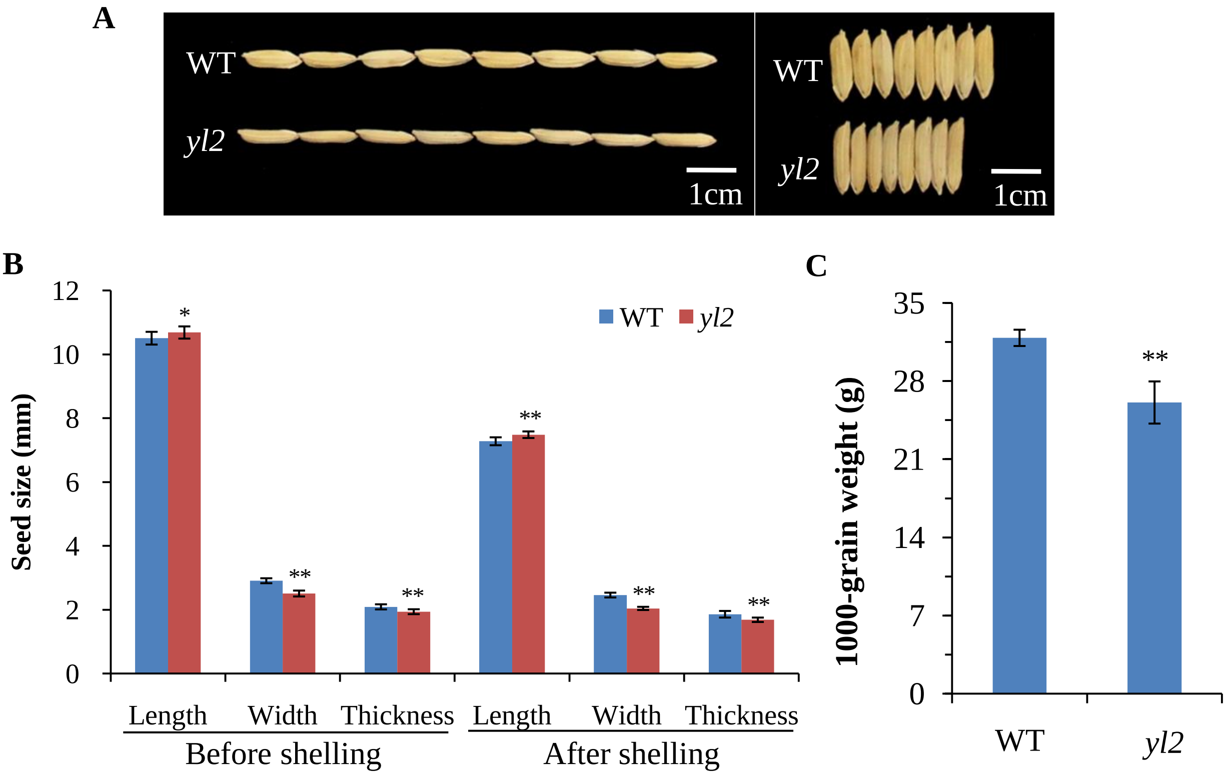

Supplementary Fig. 1 Comparison of seed size (a, b), and 1,000-grain weight (c) between the wild type (WT) and the *yl2* mutant. All data represent means ± SD ($n = 15$), *, P<0.05; **, P<0.01.

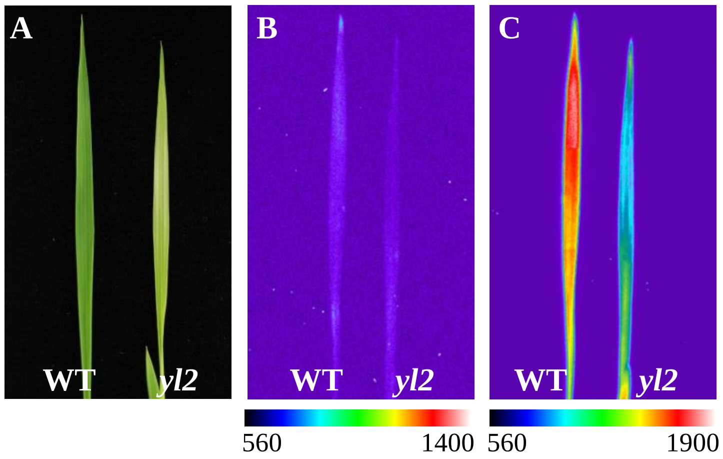

Supplementary Fig. 2 Chlorophyll autofluorescence observations of the wild-type (WT) and *yl2* mutant. Leaves from 7-day-old seedlings were collected for observation. a bright images, b dark-level chlorophyll fluorescence (*Fo*), and c fluorescence after 2 min illumination (*Fs*). The pseudocolor bar below shows the range of luminescence intensity in each image.

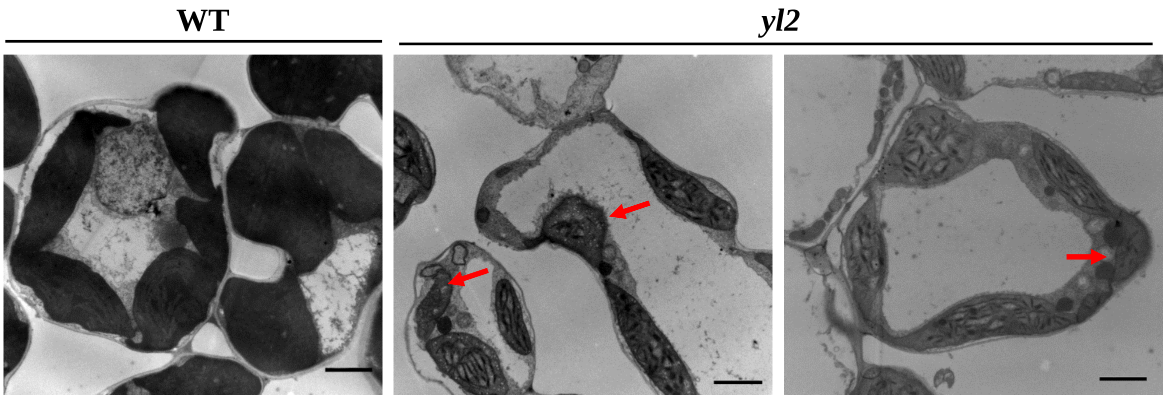

Supplementary Fig. 3 Transmission electron microscope (TEM) observations of wild-type (WT) and *yl2* mutant chloroplasts. Tissues were collected from the first fully expanded leaves of 7-day-old seedlings. WT chloroplasts had normal thylakoids and stacked membranes, while *yl2* mutant showed abnormal chloroplast development, often with less abundant thylakoid membranes (red arow). Scale bars, 2.0 µm.

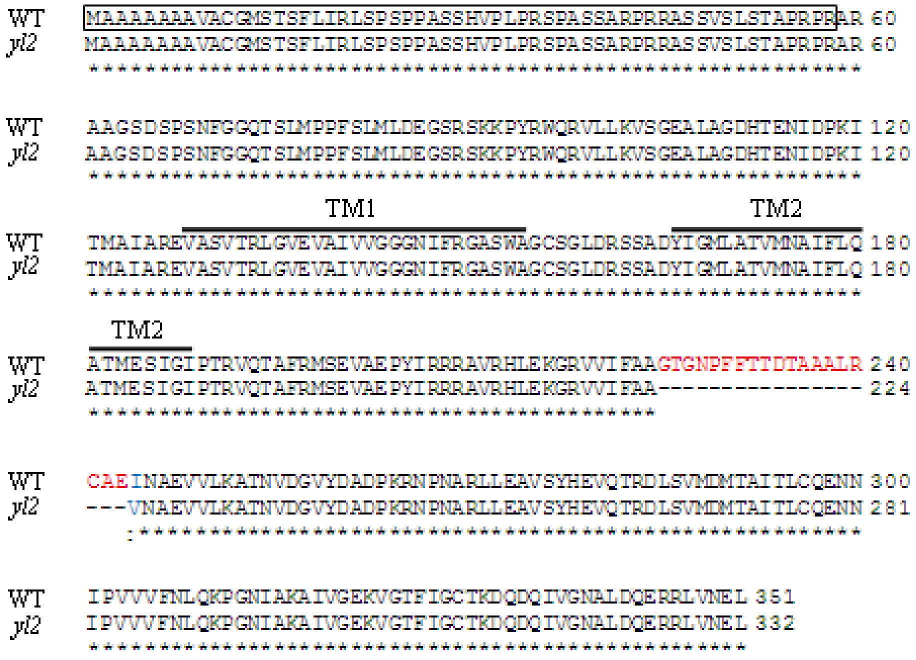

Supplementary Fig. 4 Alignment of wide-type (WT) and *yl2* mutant protein sequences. A single nucleotide substitution (G to A) in the intron-splicing site of *YL2* results in a truncated protein lacking 19 amino acids (red markings) and one amino acid residue substitution (Ile to Val, blue marking) in the *yl2* mutant. The positions of the possible chloroplast signal peptide (black box) and two possible transmembrane regions (black line; TM1 and TM2) are indicated.

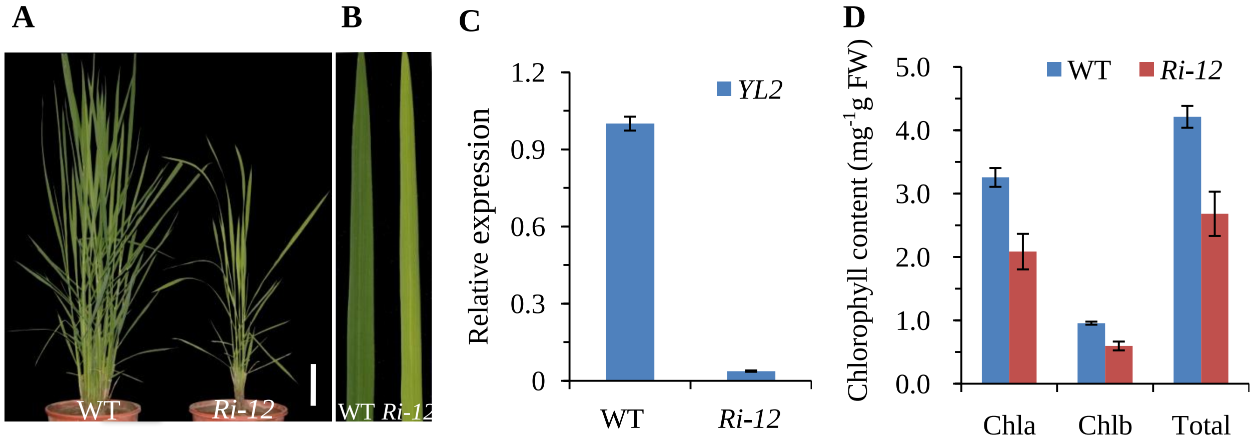

Supplementary Fig. 5 Phenotype and expression analysis of *YL2* transgenic RNAi plants. a Morphology of the wild-type (WT, Nipponbare) and transgenic line (*Ri-12*) at the booting stage. Bar: 10 cm. b Enlarged images of (a). c Relative expression levels of *YL2* in the WT and transgenic plant leaves. d Chlorophyll contents of WT and transgenic leaves at the botting stage. Data represent means ± SD (*n* = *5*). Chla: chlorophyll a, Chlb: chlorophyll b, FW: fresh weight.

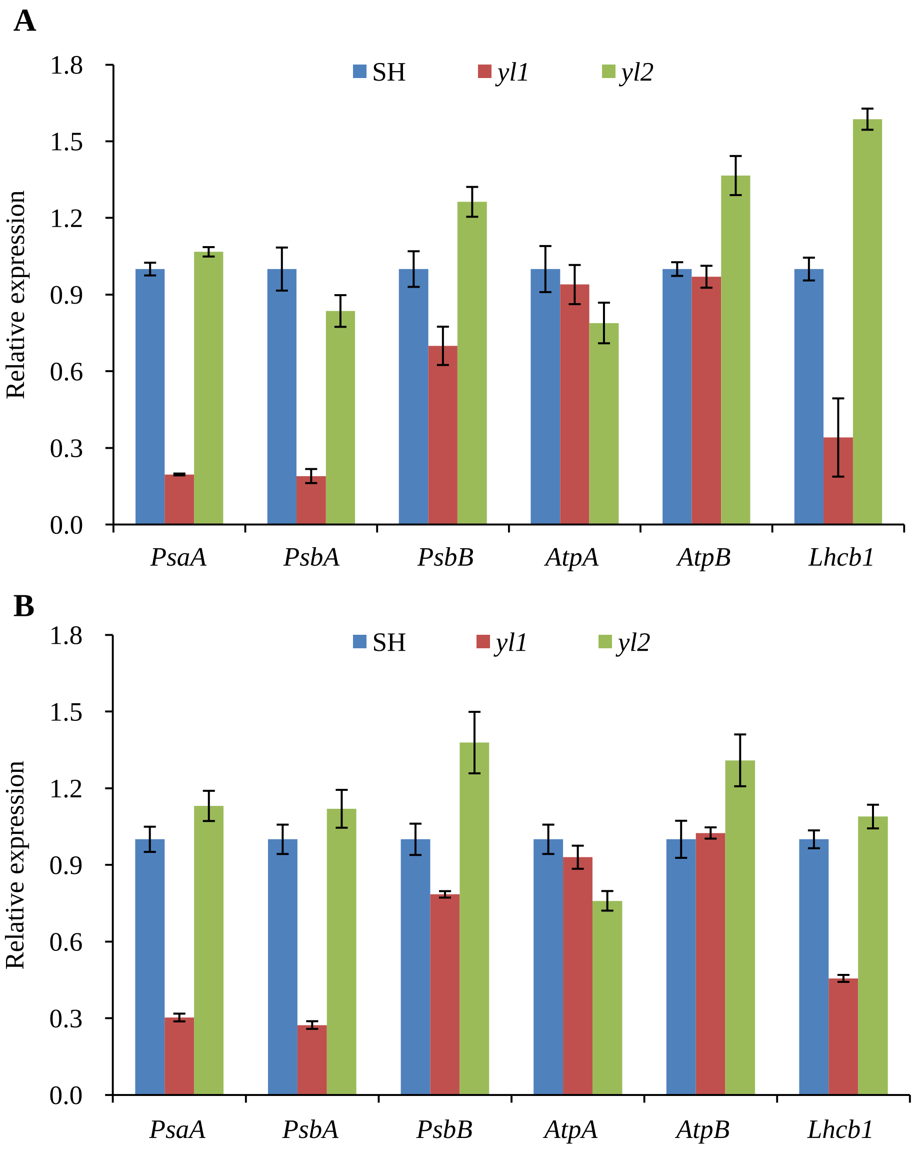

Supplementary Fig. 6 Relative expression levels of representative genes encoding thylakoid membrane proteins in leaves from 2- (A) and 4- (B) week-old WT, *yl2* and *yl1* mutants. *yl1* (*yellow leaf 1*) is another rice yellow leaf mutant reported in our previously study. In *yl1* mutant, the transcript levels of most photosynthesis associated genes were severely suppressed compared with the wild type (Chen et al., 2016). Data represent means ± SD (n = 3).

Supplementary Table 1 Protein spots identified with MALDI-TOF/TOF.

Name	NCBI No.	Mass	Score	Matches	Source
AtpA	gi|11466784	55687	412	7	Oryza sativa Japonica Group
AtpB	gi|11466794	54037	992	15	Oryza sativa Japonica Group

Supplementary Table 2 A list of primers used in this study.

Primer name	Primer sequence
Map-based cloning	
RM6321-F	GGCTCTACCTCGCTGTTGTC
RM6321-R	ACGAATATAACCTGCGGCAG
YP3248-F	GTGATGAAGACCCACGACGT
YP3248-R	CCTCTTCCTCGCGGACATG
YP3252-F	GAGCTTGATTGGGTACTGTAGC
YP3252-R	CACCCGTCCTGATATGTCCT
YP3254-F	GGATCCATGCAACTTTGGTG
YP3254-R	AGCCACCCACATTGTTTGTT
YP3258-F	TCTGCTCAGGAACGACACCT
YP3258-R	TGTATCTCCCACCGAGGCAT
YP3289-F	AAGGTAAATGATGACGTGG
YP3289-R	GGTCAATGCATACTGCTT
YP3295-F	GGAATATGGCGTGCAGACCT
YP3295-R	AACCAAGCAATGCCAGTGCT
YP3611-F	CGTCCATCTTCACCCGTCACT
YP3611-R	CGCTTGTAGTACTCCTGCTGCT
**Genotyping**	
YP3541-F	CCAACCCGTGTCCAAACTGCAT
YP3742-R	GTCTGCGTCGTACACACCGT
**Quantitive real time PCR**	
YL2-F	GGATCCATGGCCGCCGCCG
YL2-R	CGTGCCCTCGGCCTAG
V1-F	AGAATCAGCGCGAGAAGAGAACCT
V1-R	TACACCAGCTTTGGAGGAGCTGAA
V2-F	AGCAGATCCGTGATTACATGGCGA
V2-R	TGCCTCTTCACTCTCTGCAACCAA
V3-F	AACGAGAGATCTGGGCTGAATGCT
V3-R	CTCTCATTAACATGTGTTGC
RpoA-F	TAGATGCTGTATCCATGCCT
RpoA-R	CTCTTCCTCCGTGTGAAGAA

OsSig2A-F	AGTCTTATGGCATCTTGAGTG
OsSig2A-R	GACCGCTTCTTCTTTGAGG
Rps15-F	AGATACGGAGACTTGCTTCA
Rps15-R	GCTCCCTAATATCCAACTGACT
RpoTP-F	AAGCAGACAGTGATGACATC
RpoTP-R	ATCTTTGCACAATCACCAAG
Cab1-F	CCGGAGACGTTCGCCAAGA
Cab1-R	ATGAGCACCACCTGCACCG
AtpA-F	TCCAGTGGAAGAGCAGATAGCT
AtpA-R	CGTTCGAGTTGTTCCTGAATAGCT
AtpB-F	TCGCAATTCTTGGGTTGGATG
AtpB-R	TCGTGCTAGCTTCATCGATGT
PsaA-F	TAGCCTGGTTCCAAGACGTA
PsaA-R	TTTGGACCAATTCAAGGTGA
PsbA-F	AGAGACGCGAAAGTACAAGC
PsbA-R	AAGTTGCGGTCAATAAGGTA
PsbB(CP47)-F	ATGGGTTTGCCTTGGTATCGT
PsbB(CP47)-R	CTCCACATTGGATCCAGAACAGG
Lhcb1-F	CATGTTCTCCATGTTCGGC
Lhcb1-R	GACGAAGTTGGTGGCGTAG
Actin-F	CATCTTGGCATCTCTCAGCAC
Actin-R	AACTTTGTCCACGCTAATGAA
**Complementation Construct**	
pCYL2-F	ggtaccGTCGGAGCTCCTCTTCGTCG
pCYL2-R	ctgcagTGGGAGAGATGACGCGAGGT
**RNAi**	
pRNAi-F1	gagctcGACTAGGTGTAGAGGTTGCT
pRNAi-R1	actagtGCATTGATTTCTGCACAACG
pRNAi-F2	ggatccGACTAGGTGTAGAGGTTGCT
pRNAi-R2	ggtaccGCATTGATTTCTGCACAACG
**GFP Assay**	
YL2-GFP-F	caccATGGCCGCCGCCG
YL2-GFP-R	TAACTCGTTCACCAATCTTCTTTCTTGATCC
